# Exploring new lengths for *q*-ary quantum MDS codes with larger distance

**DOI:** 10.1371/journal.pone.0325027

**Published:** 2025-06-05

**Authors:** Xianmang He, Jingli Wang, Chunfang Huang, Yindong Chen

**Affiliations:** 1 Ningbo University of Finance and Economics, Ningbo, China; 2 School of Mathematics and Computer Science, Shantou University, Shantou, China; 3 Key Laboratory of Intelligent Manufacturing Technology, Ministry of Education, Shantou University, Shantou, China; Universite Cote d’Azur, FRANCE

## Abstract

In the past decade, the construction of quantum maximum distance separable codes (MDS for short) has been extensively studied. For the length $n=\frac{q^{2}-1}{m}$, where *m* is an integer that divides either *q*  +  1 or *q* − 1, a complete set of results has been available. In this paper, we dedicate to a previously unexplored cases where the length $n=\frac{q^{2}-1}{m}$, subject to the conditions that *m* is neither a divisor of *q* − 1 nor *q*  +  1. Ultimately, this problem can be summarized as exploring the necessary and sufficient conditions for the existence of pairs $\left(m_{1},m_{2}\right)$, where $m=\frac{m_{1}\times m_{2}}{m_{1}+m_{2}-2}$ is an integer, with the additional requirement that the greatest common divisor (gcd) of *m* with both *m*_1_ and *m*_2_, $\gcd(m,m_{1})>1$ and $\gcd(m,m_{2})>1$, and $\gcd(m_{1},m_{2})=2$. The quantum MDS codes presented herein are novel and exhibit distance parameters exceeding $\frac{q}{2}$.

## 1 Introduction

Quantum error-correction codes have been demonstrated as an encoding technique specifically engineered to safeguard quantum data against the effects of noise and interference. In quantum communication systems such as quantum key distribution (QKD), quantum MDS codes can be used to enhance the security and robustness of the key distribution process. They can help detect and correct errors that may occur during the transmission of quantum states used for key generation, ensuring the integrity of the shared secret key.

The construction of quantum error-correcting codes has been transformed into a finding for classical self-orthogonal codes over the fields ${𝔽}_{2}$ or ${𝔽}_{4}$ with respect to specific inner products, as referenced in [3]. This concept was later extended to the non-binary cases in [1,21]. Henceforth, the construction of quantum error-correcting codes has seen significant advancements following the realization of the interplay between quantum codes and classical codes. Let *q* be a prime power, and a *q*-ary quantum code is defined as *K*-dimensional subspace within the Hibert space $({\mathbb{C}}^{q}{)}^{⨂n}≅{\mathbb{C}}^{q^{n}}$, which is capable of detecting quantum errors at most *d* − 1. Let $k=\mathrm{\backslash textup}{log}_{q}K$, and we denote a *q*-ary quantum code as $[[n,k,d]{]}_{q}$. Similar to classical coding theory, one of the fundamental challenge in quantum coding theory is to develop quantum codes with desirable parameters. The inequality 2d≤n−k+2 provides a bound on the distance achievable for a quantum code $[[n,k,d]{]}_{q}$ (as detailed in [17,18]). A quantum code attaining this bound is referred to as a quantum maximum-distance-separable (MDS) code. Numerous classes of quantum MDS codes have been systematically constructed employing various approaches.

The Hermitian inner produce over ${𝔽}_{q^{2}}^{n}$ is defined as follows. $<u,v{>}_{h}=u_{1}v_{1}^{q}+\cdots +u_{n}v_{n}^{q}$, where $u=(u_{1},\ldots ,u_{n})$ and $v=(v_{1},\ldots ,v_{n})$ are vectors in ${𝔽}_{q^{2}}^{n}$. The approach outlined below represents a construction of *q*-ary quantum MDS codes from Hermitian self-orthogonal MDS codes over ${𝔽}_{q^{2}}^{n}$, which is one of the significant techniques presented in [1].

**Theorem 1** (*Hermitian Construction*). *Suppose that ℂ is an [[n,k,n − *k*  +  $1]{]}_{q^{2}}$ MDS code over ${𝔽}_{q^{2}}$, and is orthogonal with respect to the Hermitian inner product. Then we can construct a q-ary quantum MDS code of parameters $[[n,n-2k,k+1]{]}_{q}$*.

### 1.1 Contributions and organization

The quantum MDS codes have several potential applications: one of the most important applications is in quantum error correction, and can be used to detect and correct errors in quantum information. By using quantum MDS codes, quantum operations can be performed in a way that is resilient to certain types of errors. This is essential for building large-scale and practical quantum computers that can run complex algorithms without being overwhelmed by errors.

**Contributions**. For length of the form $\frac{q^{2}-1}{m}$, where *m* is an integer such that *m* divides *q* − 1 or *m* divides *q* + 1, comprehensive results are available, as shown in Table 1. The notation [a,b] represents the inclusive set of integers from *a* to *b*. However, in this paper, we concentrate on the case where *m* is not a divisor of either *q* − 1 or *q* + 1. Specifically, we consider pairs $\left(m_{1},m_{2}\right)$ such that $m=\frac{m_{1}\cdot m_{2}}{m_{1}+m_{2}-2}$ is an integer and $\gcd(m_{1},m_{2})=2$.

**Table 1:** Quantum MDS code with Length $\frac{q^{2}-1}{m}$.

Length	Distance	References
$\frac{q^{2}-1}{m}$, m|q+1, *m* even	$d\in [2,\frac{q-1}{2}+\frac{q+1}{m}]$	[24,25]
$\frac{q^{2}-1}{m}$, m|q+1, *m* odd	$d\in [2,\frac{q-1}{2}+\frac{q+1}{2m}]$	[4,10,24]
$\frac{q^{2}-1}{m}$, m|q−1, *m* even	$d\in [2,\frac{q+1}{2}+\frac{q-1}{m}]$	[4,10]

1. When *m* odd, m|q−1, it is clear that Hermitian self-orthogonal MDS codes can’t be constructed with the length $n=\frac{q^{2}-1}{m}$. As illustrated in Corollary 1, our construction covers some cases.
2. We provide the necessary and sufficient conditions for the existence of pairs $\left(m_{1},m_{2}\right)$ that makes $m=\frac{m_{1}\cdot m_{2}}{m_{1}+m_{2}-2}$ an integer.
3. Additionally, for any given integer $m=a_{1}b_{1}$, Algorithms 1 and 2 help us determine several possible pairs $\left(m_{1},m_{2}\right)$ such that $m=\frac{m_{1}\cdot m_{2}}{m_{1}+m_{2}-2}$ is an integer.

**Organization**. The rest part of this paper is structured as below. In Sect 2: Previous Known Results, we briefly review the previous known results about constructions for quantum MDS codes. In Sect 3: Preliminaries, we introduce the necessary preliminaries. Our main results are established in Sect 4: Constructions. The paper comes to an end in Sect 5: Conclusion.

## 2 Previous known results

The construction of quantum MDS codes has been a significant area of research since the pioneering work of Shor [23] and the subsequent generalizations by Calderbank, Rains, Shor, and Sloane [3]. These works laid the foundation for constructing quantum codes using classical codes over finite fields. The central idea is to find classical self-orthogonal codes with certain properties and then convert them into quantum codes [1,21].

An important method for constructing quantum MDS codes is the Hermitian construction. This method involves finding classical codes that are self-orthogonal with respect to the Hermitian inner product. The Hermitian construction has been used to create quantum MDS codes with larger minimum distances than those achievable through Euclidean self-orthogonal codes. For example, Jin, Ling, Luo, and Xing have used classical Hermitian self-orthogonal MDS codes to construct quantum MDS codes [12,13]. Additionally, Kai and Zhu have developed new quantum MDS codes from negacyclic codes [15], and Zhang and Chen have introduced new quantum MDS codes with large minimum distances [4]. Xueying Shi, Qin Yue and Xiaomeng Zhu use the classical Hermitian self-orthogonal generalized Reed-Solomon codes to construct some new quantum MDS codes with minimum distance bigger than $\frac{q}{2}$  +  1 [22]. Reference [6] constructs six new classes of *q*-ary quantum MDS codes by using generalized Reed-Solomon (GRS) codes and Hermitian construction.

Generalized Reed-Solomon (GRS) codes have been particularly useful in constructing quantum MDS codes. GRS codes are a generalization of the classical Reed-Solomon codes and are known for their optimal error-correcting capabilities. By using GRS codes, several new classes of quantum MDS codes have been constructed with parameters that exceed the minimum distance of previously known codes. For example, Reference [20] constructs a new family of quantum MDS codes from classical generalized Reed-Solomon codes and derive the necessary and sufficient condition for the existence. Jin [14] presents a new construction of quantum MDS codes with minimum distance greater than $\frac{q}{2}$  +  1. The authors use Hermitian self-orthogonal codes to construct these new quantum MDS code. Reference [2] constructs quantum MDS codes with parameters $[[q^{2}+1,q^{2}+3-2d,d]{]}_{q}$ for all d≤q+1, d≠q. These codes are shown to exist by proving that there are classical generalised Reed-Solomon codes which contain their Hermitian dual.

Constacyclic codes over finite fields have been another rich source for constructing quantum MDS codes. These codes offer a flexible structure that can be tailored to achieve the MDS property. Kal *et al*. [16] generated several classes of quantum MDS codes based on constacyclic codes. Subsequently, Chen *et al*. [4] got four families of *q*-ary quantum MDS codes through MDS cyclic codes. Hu *et al*. [11] proposed a way to determine the maximum distance of $[[n,k,d]{]}_{q}$ quantum MDS codes from constant cyclic codes with the given *n* and *q*, and in the meanwhile presented a new class of quantum MDS codes derived from Hermitian dual-containing MDS constacyclic code. Kai and Zhu [15] construct two families of quantum MDS codes by leveraging negacyclic codes.

In recent years, a plenty of quantum MDS codes possessing favorable properties have been derived from classical error-correcting codes, including algebraic-geometric codes, BCH codes, and Reed-Muller codes, as detailed in References [5,7,14,13,12] etc. The underlying principle of constructing the Hermitian self-orthogonal codes hinges on the solvability in ${𝔽}_{q}$ of a system of homogenous equations over ${𝔽}_{q^{2}}$ [14]. By applying Hermitian self-orthogonal algebraic geometry codes to quantum codes, some good quantum codes were obtained [13]. Grassl *et al*. [9] constructed a class of *q*-ary quantum MDS codes with length *n* = *q*^2^ − 1. La Guardia [19] constructed a class of quantum MDS codes utilizing MDS cyclic codes. By identifying polynomials rooted in appropriate trace functions, a novel family of linear codes was introduced, facilitating the construction of stabilizer quantum codes over several finite fields [8].

## 3 Preliminaries

In this section, we introduce a straightforward approach to constructing generator matrices that are crucial for the formation of Hermitian self-orthogonal MDS codes over the finite field ${𝔽}_{q^{2}}$ as detailed in Reference [10]. This approach not only recaptures the case where the code length $n=\frac{q^{2}-1}{m}$, but also paves the way for the development of a variety of new MDS quantum codes. We will proceed with two key lemmas that are fundamental to our construction. However, we choose to omit the proofs of these lemmas and instead, direct the interested reader to Reference [10] for a comprehensive explanations of the proofs.

**Lemma 2.** *Let θ be a primitive element within the multiplicative group of the finite field ${𝔽}_{q^{2}}^{*}$, and an integer m|q^2^−1, then $\sum_{j=1}^{\frac{q^{2}-1}{m}}\theta^{jtm}=0$ except the case that $t|\frac{q^{2}-1}{m}$.*

**Lemma 3.** *Let $v_{0},\ldots ,v_{n}$ be n non-zero elements in the multiplicative group ${𝔽}_{q^{2}}^{*}$. Let $gl=(g_{1l},\ldots ,g_{nl})$ for l=1,…,k be k linear independent rows in ${𝔽}_{q^{2}}^{n}$ such that $\sum_{j=1}^{n}v_{j}g_{jl_{1}}g_{jl_{2}}^{q}=0$ for any two indices l_1_ and l_2_ in the set {1, ..., k} (where $l_{1}=l_{2}$ is allowed). Consequently, we can construct a Hermitian self-orthogonal $[n,k{]}_{q^{2}}$ code produced by these k rows.*

With Lemmas 2 and 3, given m,q, and *m*|*q*^2^ − 1, for any fixed positive integer *k*, a linear error codes of length $\frac{q^{2}-1}{m}$ over ${𝔽}_{q^{2}}^{n}$ can be defined as follows:


$$\mathbb{C}=\{(\theta^{m}f(\theta^{m}),\theta^{2m}f(\theta^{2m}),\ldots ,\theta^{jm}f(\theta^{jm}),\ldots ,\theta^{(\frac{q^{2}-1}{m}-1)m}f(\theta^{(\frac{q^{2}-1}{m}-1)m}),f(1))\;:f\in {𝔽}_{q^{2}}[x],\deg(f)\leq k-1\}\;$$

It is evident that ℂ is an MDS code with the parameters $[n=\frac{q^{2}-1}{m},k,n$ − *k*  +  1] over ${𝔽}_{q^{2}}$. Essentially, this code is an evaluation code at the points $\theta^{m},\theta^{2m},\ldots ,\theta^{(\frac{q^{2}-1}{m}-1)m},1$.

The Hermitian inner product of any two codewords (associated with two polynomials *f* and *g*) is $\sum_{j=1}^{\frac{q^{2}-1}{m}}\theta^{jm+jqm}fg^{q}(\theta^{jm})$. Thus, if the sum $\sum_{j=1}^{\frac{q^{2}-1}{m}}\theta^{jm(1+q+t_{1}+t_{2}q)}=0$, where $\forall t_{1},t_{2}\in \mathrm{\backslash break}\;\mathrm{-4pt}[0,k-1]$, then ℂ is a Hermitian self-orthogonal MDS code.

## 4 Constructions

This section focuses on the construction of novel quantum MDS codes with a length of $\frac{q^{2}-1}{m}$, where m∤q − 1 and m∤q  +  1, pairs $\left(m_{1},m_{2}\right)$ with $m=\frac{m_{1}\cdot m_{2}}{m_{1}+m_{2}-2}$, $\gcd(m_{1},m_{2})=2$, and *q* represents an odd prime power.

Let $m_{1},m_{2}$ be two even integers. *m*_1_|*q* − 1, *m*_2_|*q*  +  1. According to Lemma 3.1 in [10], we have the following identity when $0\leq t_{1},t_{2}\leq \frac{q-1}{2}+\frac{q-1}{m_{1}}-1$.


$$\sum_{j=1}^{\frac{q^{2}-1}{m_{1}}}\theta^{jm_{1}(t_{1}+t_{2}q)}\cdot \theta^{j\frac{m_{1}(q+1)}{2}}=0.$$

According to Theorem 1 from Reference [25], the subsequent identity is established when $0\leq t_{1},t_{2}\leq \frac{q-1}{2}+\frac{q+1}{m_{2}}-2$:


$$\sum_{j=1}^{\frac{q^{2}-1}{m_{2}}}\theta^{jm_{2}(t_{1}+t_{2}q)}\cdot \theta^{jm_{2}(q+1)}=0.$$

By summing the two identities, we derive the following new identities:


$$\sum_{j=1}^{\frac{q^{2}-1}{m_{2}}}\theta^{jm_{2}(t_{1}+t_{2}q)}\cdot \theta^{jm_{2}(q+1)}+H(\sum_{j=1}^{\frac{q^{2}-1}{m_{1}}}\theta^{jm_{1}(t_{1}+t_{2}q)}\cdot \theta^{j\frac{m_{1}(q+1)}{2}})=0.$$

Here, *H* can be any nonzero element in ${𝔽}_{q}^{*}$, and the common position $t_{1},t_{2}$ are in the range $0\leq t_{1},t_{2}\leq \frac{q-1}{2}+\min\{\frac{q+1}{m_{2}}-2,\frac{q-1}{m_{1}}-1\}$.

Let *M* be the set $\left\{\theta^{jm_{1}}:j=1,\ldots ,\frac{q^{2}-1}{m_{1}}\right\}$ ∪ $\left\{\theta^{jm_{2}}:j=1,\ldots ,\frac{q^{2}-1}{m_{2}}\right\}$, and the code is the set $\{(f(x){)}_{x\in M}:0\leq \deg(f)\leq \frac{q-1}{2}+\min\{\frac{q+1}{2m_{2}}-2,\frac{q-1}{m_{1}}-1\}.$

**Theorem 4.** [25] *Let q be an odd prime power, m_1_,m_2_ be two even integers. $m_{1}|q-1,m_{2}|q+1$, then we construct a q-ary quantum MDS code with the following parameters:*

1. length *n*: $\frac{q^{2}-1}{m_{1}}+\frac{q^{2}-1}{m_{2}}-\frac{q^{2}-1}{\text{lcm}(m_{1},m_{2})}$, where $\text{lcm}(m_{1},m_{2})$ denotes the least common multiple of $m_{1},m_{2}$.
2. minimum distance *d*: $2\leq d\leq \frac{q-1}{2}+\min\{\frac{q+1}{m_{1}},\frac{q-1}{m_{2}}\}$.

In Theorem 4, by carefully selecting specific parameters for the pair $\left(m_{1},m_{2}\right)$, we can derive a novel class of quantum codes that exhibit highly beneficial properties, as demonstrated in Corollary 1.

**Corollary 1.** *With the notation defined as above, m_1_ = 2k is an even divisor of q − 1, $m_{2}=2k-2\geq 3$ is an even divisor of q + 1, then we can construct an q-ary quantum MDS code with the parameters:*

1. length *n*: $\frac{q^{2}-1}{k}=\frac{q-1}{k}\cdot (q+1)$,
2. minimum distance *d*: $d\in [2,\frac{q-1}{2}+\frac{q+1}{2k-2}]$.

**Table 2:** New Quantum MDS code with $n=\frac{q-1}{k}\cdot (q+1)$.

$\left(m_{1},m_{2}\right)$	q	m	Distance d
(8,6)	17	4	[2,11]
(8,6)	41	4	[2,27]
(10,8)	71	5	[2,44]
(12,10)	49	6	[2,29]
(14,12)	71	7	[2,31]
(16,14)	97	8	[2,56]
(18,16)	127	9	[2,71]

**Remark 1.** When *q* odd, k|q − 1, it is clear that Hermitian self-orthogonal MDS codes can’t be constructed by the generator matrices over the finite field ${𝔽}_{q^{2}}$ as detailed in [10]. However, this case is partially covered by Corollary 1.

Theorem 4 provides the theoretical result of the construction, but it must be ensured that $m=\frac{m_{1}m_{2}}{m_{1}+m_{2}-\gcd(m_{1},m_{2})}$ is an integer. This allows us to choose certain values pair $\left(m_{1},m_{2}\right)$ such that m∤q − 1,m∤q  +  1, but gcd(m,q − 1)>1,gcd(m,q  +  1)>1. This case has not been systematically discussed and studied.

**Lemma 5.** *Let $m_{1},m_{2}$ be two even integers, and $\gcd(m_{1},m_{2})=2$. If $m=\frac{m_{1}\times m_{2}}{m_{1}+m_{2}-2}$ is an integer, $\gcd(m_{1},m)>1,\gcd(m_{2},m)>1$, then, at least one of $m_{1}\text{and}m_{2}$ has a factorization with at least three prime factors.*

*Proof:* The proof can be accomplished by introducing the method of proof by contradiction. Without loss of generality, we can assume that $m_{1}=2a_{1}$, $m_{2}=2b_{1}$, both $a_{1}\text{and}b_{1}$ are primes, otherwise, either $m_{1}\text{or}m_{2}$ has three factors. Hence, we have $m=\frac{2a_{1}\cdot 2b_{1}}{2a_{1}+2b_{1}-2}$. Note that $m=4,2a_{1}\text{or}2b_{1}$, then $a_{1}b_{1}=a_{1}+b_{1}-1,b_{1}=a_{1}+b_{1}-1\text{or}a_{1}=a_{1}+b_{1}-1$, which is completely impossible. Therefore, $m_{1}\text{or}m_{2}$ has at least three prime factors. ◻

**Remark 2.** Consider that $\gcd(m_{1},m_{2})=2$, $m_{1},m_{2}$ even, therefore, the factorization of *m*_1_ or *m*_2_, one with one factor 2 and the other can have multiple factors 2. We can assume that $m_{1}=2a_{1}a_{2}$, $a_{1},a_{2}$ odd, $m_{2}=2b_{1}b_{2}$, *b*_1_,*b*_2_ can be odd or even, otherwise, $\gcd(m_{1},m_{2})\neq 2$.

In the following text, assume that $m_{1}=2a_{1}a_{2}$, $a_{1},a_{2}\geq 3$ odd, $m_{2}=2b_{1}b_{2}$, $b_{1},b_{2}\geq 2$, $\gcd(a_{1}a_{2},b_{1}b_{2})=2$, $m=\frac{2a_{1}a_{2}\cdot 2b_{1}b_{2}}{2a_{1}a_{2}+2b_{1}b_{2}-2}$.

**Theorem 6.** *The necessary and sufficient conditions for the existence of $\left(m_{1},m_{2}\right)$ pairs is $m_{1}\text{or}m_{2}$ has at least three prime factors. Here, $m=\frac{m_{1}\times m_{2}}{m_{1}+m_{2}-2}$ is an integer and $\gcd(m,m_{1})>1$ and $\gcd(m,m_{2})>1$*.

*Proof:* From Lemma 5, we know that $m_{1}\text{or}m_{2}$ has at least three prime factors. Next, we just need to prove that the pairs $\left(m_{1},m_{2}\right)$ always exists as long as $m_{1}=2a_{1}a_{2},m_{2}=2b_{1}b_{2}$.

Let $m=\frac{2a_{1}a_{2}\cdot 2b_{1}b_{2}}{2a_{1}a_{2}+2b_{1}b_{2}-2}=2a_{1}b_{1}$, then we have $2a_{1}a_{2}+2b_{1}b_{2}-2=2a_{2}b_{2}$, then $b_{2}(a_{2}-b_{1})=a_{1}a_{2}-1$.

With the assumption that $a_{1},a_{2}$ are odd, therefore, $a_{1}a_{2}-1$ can be factorised into $p_{1}p_{2}$, that is, $b_{2}(a_{2}-b_{1})=a_{1}a_{2}-1=p_{1}p_{2}$. Hence, let $b_{2}=p_{1},a_{2}-b_{1}=p_{2}$ or $b_{2}=p_{2},a_{2}-b_{1}=p_{1}$ to make the equation holds.

Now, we need to prove that $a_{2}-p_{1}\geq 2$. From the equation $a_{1}a_{2}=p_{1}p_{2}+1$, and $a_{1},a_{2}$ odd, then we have $a_{2}(a_{1}-2)\geq p_{1}^{2}+1$. $a_{2}^{2}-2a_{2}-p_{1}^{2}-1\geq 0$, $(a_{2}-1{)}^{2}\geq p_{1}^{2}+2$, $a_{2}-1\geq \sqrt{p_{1}^{2}+2}>p_{1}+\sqrt{2}$. $a_{2}-p_{1}>1+\sqrt{2}$. Consider that $a_{2},p_{1}$ are integers, therefore, $a_{2}\geq p_{1}+2$.

The conclusion holds. ◻

**Remark 3.** Theorem 6 provides an existence case, and other cases can be similarly proved. For example, when *m*_1_ = 2*p*, *p* odd. Even if *p* a prime, we can also prove its existence. Let $m_{2}=2b_{1}b_{2}$, $b_{1},b_{2}\geq 2$ integers. Similarly, let *m* = 2*b*_1_, then we have $pb_{2}=p+b_{1}b_{2}$ − 1, (p − $b_{1})b_{2}=p$ − 1. Hence, let $b_{1}=\frac{p+1}{2},b_{2}=2$ to make the equation holds. We treat this case in the following Algorithm 1.

**Algorithm 1.** Algorithm for determining parameters $m_{1},m_{2}$.



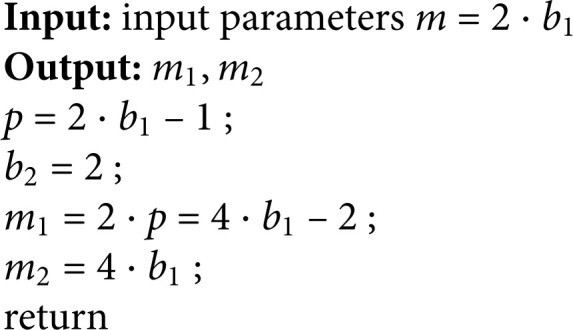



**Remark 4.** Theorem 6 demonstrates that given an arbitrary *m*_1_, *m*_2_ can always be found such that $\frac{m_{1}m_{2}}{m_{1}+m_{2}-2}$ is an integer. Similarly, given *m*_2_, *m*_1_ can also be found such that $\frac{m_{1}m_{2}}{m_{1}+m_{2}-2}$ is an integer. Considering that this proof is identical, we omit here. The prerequisite for this is that *m*_1_ or *m*_2_ has at least three prime factors.

### Algorithm to choose the parameters $m_{1},m_{2}$

Theorem 6 only tells us the existence of the pair $\left(m_{1},m_{2}\right)$, we need to fully determine the value of a pair $\left(m_{1},m_{2}\right)$. Given any integer $m=a_{1}b_{1}$, Algorithm 2 can help us determine several possible pairs $\left(m_{1},m_{2}\right)$.

According to the proof process of Theorem 6, the algorithm run through the variable *p*_1_, which varies from 1 to $a_{1}b_{1}$ − 1 or larger, which can be used to determine the pair $\left(m_{1},m_{2}\right)$. From Corollary 2, $p_{2}\in Z$ always exists.

**Corollary 2.** *Given two integer $a_{1}\geq 2$ odd and $b_{1}\geq 1$ , $m=2\cdot a_{1}\cdot b_{1}$, there exists a pair $\left(p_{1},p_{2}\right)$ that makes $m=\frac{m_{1}m_{2}}{m_{1}+m_{2}-2}\in Z$, Here, $p_{2}=\frac{a_{1}(b_{1}+p_{1})-1}{p_{1}}$, $m_{1}=2a_{1}(b_{1}+p_{1}),m_{2}=2b_{1}p_{2}$*.

*Proof:* Assume that $p_{1}=a_{1}b_{1}-1$, then $p_{2}=\frac{a_{1}(b_{1}+p_{1})-1}{p_{1}}=1+a_{1}\geq 2\in Z$. The corollary holds. ◻

**Algorithm 2.** Algorithm for determining parameters $m_{1},m_{2}$.



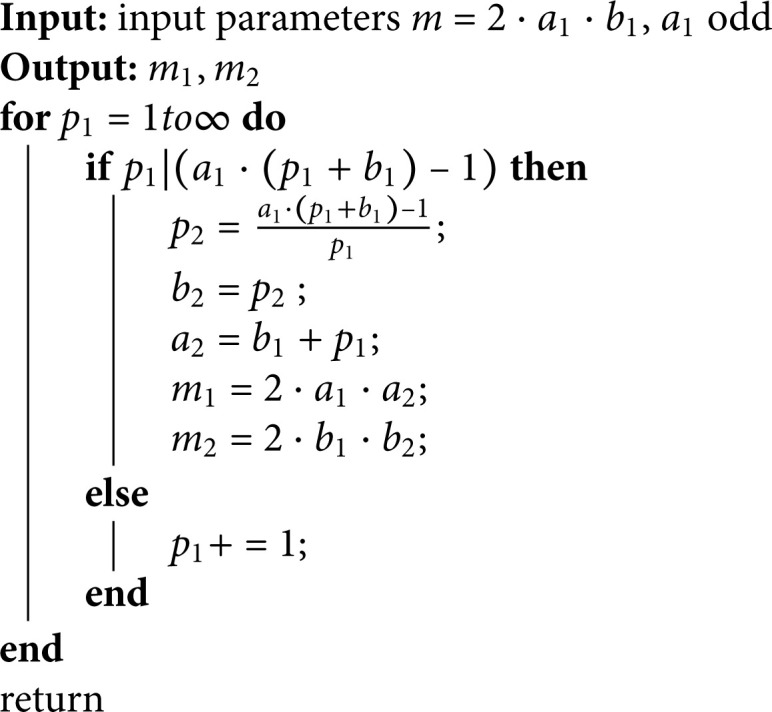



With Algorithm 1, we can get the value of pairs $\left(m_{1},m_{2}\right)$. Let’s make some examples to illustrate this.

**Example 1.** Let $m=2a_{1}b_{1}=2\cdot 3\cdot 4$, $p_{1}=a_{1}\cdot b_{1}-1=11$, then $m_{1}=2\cdot 3\cdot (4+11)=90,m_{2}=2\cdot 4\cdot \frac{a_{1}\cdot (p_{1}+b_{1})-1}{p_{1}}=32$. Let *p*_1_ = 1 also can give a pair of *m* = 24, $b_{2}=\frac{a_{1}\cdot (p_{1}+b_{1})-1}{p_{1}}=7$, $m_{1}=30,m_{2}=112$.

**Example 2.** Let $m=2a_{1}b_{1}=2\cdot 3\cdot 5$, $p_{1}=1,b_{2}=17$, then $m_{1}=36,m_{2}=170$. Case *p* = 2 can also give a pair $\left(m_{1},m_{2}\right)$ with *m* = 30, $m_{1}=42,m_{2}=100$. Let *p*_1_ = 7, $m_{1}=72,m_{2}=50$. If *p* = 14, $m_{1}=113,m_{2}=40$.

**Example 3.** Let $m=2a_{1}b_{1}=2\cdot 5\cdot 4$, *p* = 1, then $m_{1}=50,m_{2}=192.$ Let *p*_1_ = 19, then $m_{1}=230,m_{2}=48$.

Now, we need to determine the *q* that makes $m=a_{1}b_{1},m_{1}|q-1,m_{2}|q+1,m∤q-1,m∤q+1$. Consider that $\gcd(m_{1},m_{2})=2$, then there exists two integers $l_{0},k_{0}$ fulfilling $l_{0}m_{1}+2=k_{0}m_{2}$. Set $q=m_{1}m_{2}t+l_{0}m_{1}+1$, or $q=m_{1}m_{2}t+k_{0}m_{2}-1$ and it is easy to verify that $m_{1}|q-1,m_{2}|q+1.$ Table 3 gives some examples of new quantum MDS codes, with the length $n=\frac{q^{2}-1}{m}$, but m∤q−1, m∤q+1.

**Table 3:** New Quantum MDS code with $n=\frac{q^{2}-1}{m},m∤q-1,m∤q+1$.

$\left(m_{1},m_{2}\right)$	q	m	Distance d
(30,112)	449	24	228
(90,32)	449	24	229
(18,32)	127	12	66
(42,16)	127	12	66
(50,32)	449	20	233
(30,56)	449	20	232
(110,24)	769	20	391
(40,114)	1481	30	752

**Remark 5.** The integer 2 plays a special role throughout the entire paper, including $\gcd(m_{1},m_{2})=2$, $m=\frac{m_{1}\cdot m_{2}}{m_{1}+m_{2}-2}$. The reason for doing this is to consider the existence of *q*, which only exists when $\gcd(m_{1},m_{2})=1$ or 2, and satisfies *m*_1_|*q*−1, *m*_2_|*q* + 1 in the meanwhile.

## 5 Conclusion

In this paper, we have conducted a investigation of the case where the length is given by $n=\frac{q^{2}-1}{m}$, under the conditions that m∤q−1,m∤q+1, $m=\frac{m_{1}m_{2}}{m_{1}+m_{2}-2}$, where $m_{1}\text{and}m_{2}$ are both even, and $\gcd(m_{1},m_{2})=2$. We have derived the necessary and sufficient conditions for the existence of such pairs $\left(m_{1},m_{2}\right)$. Additionally, for a specified value of *m*, we design Algorithms 1 and 2 to determine the pair $\left(m_{1},m_{2}\right)$. With these insights, it is now a straightforward task to construct a new class of quantum MDS codes.

Despite the numerous methods have been proposed to construct quantum MDS codes, in fact, the code length *n* is still sparse for $q\leq n\leq q^{2}$. In most cases, codes have not been constructed because the majority of the constructed results are concentrated in the case of $n=\frac{q^{2}-1}{m}$. Our future work is to develop a general method that is not limited to the case of length $n=\frac{q^{2}-1}{m}$.
